# The Intersection of CMOS Microsystems and Upconversion Nanoparticles for Luminescence Bioimaging and Bioassays

**DOI:** 10.3390/s140916829

**Published:** 2014-09-10

**Authors:** Liping. Wei, Samer. Doughan, Yi. Han, Matthew V. DaCosta, Ulrich J. Krull, Derek. Ho

**Affiliations:** 1 Department of Physics and Materials Science, City University of Hong Kong, 83 Tat Chee Avenue, Kowloon, Hong Kong; E-Mail: weiliping1988@gmail.com; 2 Department of Chemical and Physical Sciences, University of Toronto Mississauga, 3359 Mississauga Road North, Mississauga, Ontario L5L 1C6, Canada; E-Mails: samer.doughan@utoronto.ca (S.D.); yi.han@utoronto.ca (Y.H.); matt.dacosta@utoronto.ca (M.V.D.); ulrich.krull@utoronto.ca (U.J.K.)

**Keywords:** upconversion nanoparticle, CMOS, bioimaging, bioassays, biosensors, luminescence resonance energy transfer, fluorescence, multiplexing

## Abstract

Organic fluorophores and quantum dots are ubiquitous as contrast agents for bio-imaging and as labels in bioassays to enable the detection of biological targets and processes. Upconversion nanoparticles (UCNPs) offer a different set of opportunities as labels in bioassays and for bioimaging. UCNPs are excited at near-infrared (NIR) wavelengths where biological molecules are optically transparent, and their luminesce in the visible and ultraviolet (UV) wavelength range is suitable for detection using complementary metal-oxide-semiconductor (CMOS) technology. These nanoparticles provide multiple sharp emission bands, long lifetimes, tunable emission, high photostability, and low cytotoxicity, which render them particularly useful for bio-imaging applications and multiplexed bioassays. This paper surveys several key concepts surrounding upconversion nanoparticles and the systems that detect and process the corresponding luminescence signals. The principle of photon upconversion, tuning of emission wavelengths, UCNP bioassays, and UCNP time-resolved techniques are described. Electronic readout systems for signal detection and processing suitable for UCNP luminescence using CMOS technology are discussed. This includes recent progress in miniaturized detectors, integrated spectral sensing, and high-precision time-domain circuits. Emphasis is placed on the physical attributes of UCNPs that map strongly to the technical features that CMOS devices excel in delivering, exploring the interoperability between the two technologies.

## 1. Introduction

Medical, environmental, and life science applications generate demands for low-cost small-size biochemical assay systems. These systems consist of bioassay chemistry and readout electronics responsible for analytical signal transduction and subsequent processing, respectively. The use of organic fluorophores as contrast agents for bio-imaging when coupled with appropriate selective biorecognition molecules enables visualization of biological processes. Although optical bio-imaging and bioassays that make use of fluorophores are common, these applications are not without limitations. Molecular fluorophores have broad emission spectra that render them unsuitable for implementation for higher densities of multiplexed biolabeling. They also often suffer from low sensitivity and photodegradation. Quantum dots that feature a large molar extinction coefficient, high quantum yield, narrow emission bandwidth, size-dependent tunable emission and high photostability are attractive as alternative fluorescent labels for optical labeling and imaging. However, the use of quantum dots for biological detection is limited by several factors. The potential toxicity of quantum dots that may pose risks to human health and the environment is a major drawback. Intermittent emission also limits their use for labeling individual biological molecules. In addition, both the organic fluorophores and quantum dots are generally excited with ultraviolet (UV) and visible light. Absorption of UV and visible light by the biological samples often induces autofluorescence, which interferes with fluorescent signals obtained from labeled exogenous biomarkers. Prolonged exposure of the biological samples to UV radiation can also cause sample photodamage and mutations [1].

The drawbacks of the Stokes-shifting dyes and quantum dots in biological applications have prompted the development of a new class of lanthanide-doped nanomaterials termed upconversion (UC) nanoparticles. UC nanoparticles, or UCNPs, exhibit anti-Stokes emission, and can be excited in the near-infrared (NIR) spectral region where biological molecules are generally optically transparent. In addition, these nanoparticles typically show sharp emission bands at a number of wavelengths, long lifetimes, tunable emission, high photostability, and low cytotoxicity, which render them particularly useful for bio-imaging and bioassay applications [1].

This paper surveys several key concepts surrounding upconversion nanoparticles and the systems that detect and process the luminescence signal. It is organized as follows. Section 2 describes the principle of photon upconversion, tuning of emission wavelengths, UCNP bioassays, and UCNP time-resolved techniques. Section 3 surveys the electronic readout system for signal detection and processing suitable for UCNP luminescence. The section describes the evolution and recent progress in miniaturized detectors, integrated spectral sensing, and high-precision time-domain circuits implemented in the complementary metal-oxide-semiconductor (CMOS) technology. Section 4 highlights key characteristics that result from combination of UCNPs and CMOS technology in a system. Throughout the paper, emphasis is placed on the physical attributes of UCNPs that map strongly to the technical features that CMOS devices excel in delivering, exploring the synergy between the two technologies.

## 2. Upconversion Nanoparticles

Advances in the ability to design the emission wavelength with a high degree of precision are the main driver for the recent interest in adoption of UCNPs. In this section, the principle of photon upconversion, optical tuning of the emission spectra of UCNPs, their application in bioassays, and their suitability for interrogation using time-resolved techniques are described.

### 2.1. Photon Upconversion

In contrast to conventional Stokes-type luminescence processes that are defined by a single ground- and a single excited-state, the UC process generates anti-Stokes emissions via the sequential absorption of two or more low-energy excitation photons through multiple, long-lived, metastable excited states. The various processes that lead to photon UC have recently been thoroughly reviewed, and the interested reader is directed elsewhere [1,2]. The anti-Stokes process is of interest as a mechanism to provide an analytical signal that is suitable for the development of optical assays and sensor technologies. The potential for excitation using low energy photons that are not strongly absorbed or scattered by biological and CMOS materials enables minimization of optical background interference, while the emission process can provide a wide spectral separation from the excitation wavelength.

Trivalent rare-earth lanthanide ions (Ln^3+^) have been implemented as dopants in various inorganic crystalline hosts such as NaYF_4_ to fabricate luminescent nanoparticles (NPs) with unique optical properties. The various synthetic strategies employed to fabricate these materials in a controlled fashion have recently been reviewed [1]. Such lanthanide doped NPs can exhibit optical down conversion (DC) as well as UC properties. DC refers to Stokes shifted emission where photons of lower energy are emitted, typically after excitation with radiation at the UV/blue part of the spectrum. UC refers to the anti-Stokes process where NPs absorb long-wavelength excitation radiation in the infrared (IR) or near-infrared region of the electromagnetic spectrum and emit photons of shorter wavelength, typically in a broad wavelength regime from UV to IR. The latter NPs are termed upconverting nanoparticles (UCNPs). Ln^3+^ ions are ideal candidates for photon UC since they possess long lived, metastable intermediate energy levels that are equally spaced and oriented in a ladder-type arrangement. The absorption and emission spectra of Ln^3+^ ions arise from Laporte forbidden 4f-4f electronic transitions. The shielding of these 4f electrons by the filled 5s^2^ and 5p^6^ subshells results in very narrow absorption and emissions bands from nanoparticles doped with these trivalent species [1]. Weak coupling between the crystal field of the host lattice and the f-f transition of the lanthanide ion means the emissions generated from a given lanthanide ion is nearly independent of the host material selected [3]. A wide variety of Ln^3+^ ions have been implemented in DC and UC systems [1]. Due to the involvement of spin-forbidden electronic transitions and anti-Stokes emissions, most lanthanide emissions should be more correctly referred to as luminescence rather than as fluorescence.

### 2.2. UCNP Structure and Optical Properties

**Figure 1.**
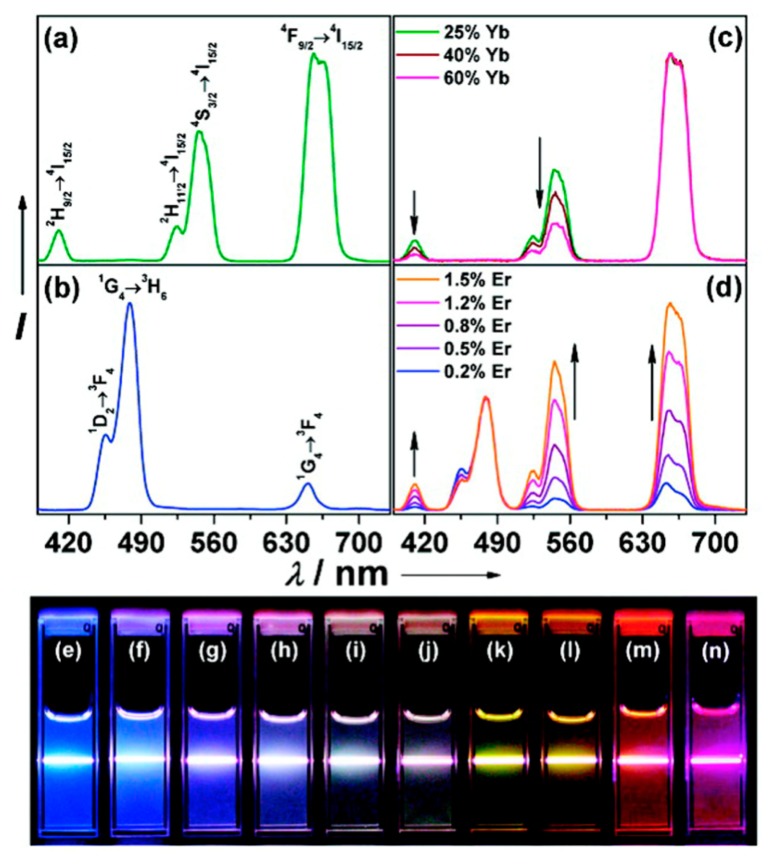
Emission spectra of (**a**) NaYF_4_: Yb, Er (18, 2 mol%), (**b**) NaYF_4_: Yb, Tm (20, 0.2 mol%), (**c**) NaYF_4_: Yb, Er (25–60, 2 mol%), and (**d**) NaYF_4_: Yb, Tm, Er (20, 0.2, 0.2–1.5 mol%) UCNPs in ethanol solution at 10 mM concentrations. Emissions spectra depicted in (c) and (d) have been normalized to Er^3+^ 650 nm and Tm^3+^ 480 nm emissions, respectively. (**e**), (**f**–**j**), and (**k**–**n**) are compiled luminescent photos of colloidal solutions of NaYF_4_: Yb, Tm (20, 0.2 mol%), NaYF_4_: Yb, Tm, Er (20, 0.2, 0.2–1.5 mol%), and NaYF_4_: Yb, Er (18–60, 2 mol%), respectively, upon 980 nm excitation using a 600 mW diode laser [5].

Luminescent UCNPs are typically comprised of an inorganic crystalline host material into which Ln^3+^ ions are embedded at low concentrations. The inorganic host is optically transparent and serves primarily as a scaffold orienting the optically active dopants appropriately in three-dimensional space. The architecture of UCNPs is typically in the form of core only, core-shell, or nanocomposite structures, each offering the potential to tune the optical properties of the resultant material. While many Ln^+3^ doped materials may exhibit NIR sensitized UC emissions, efficient and tunable UC luminescence is realized only for certain materials. Considerations for efficient UC emission in terms of architecture, host lattice choice, dopant ion selection, and dopant concentration have recently been presented [1]. In general, combinations of different lanthanide ions within the same material allows for some flexibility in the selection of emission wavelengths from an UCNP, due to the unique energy level structure of each lanthanide ion. By manipulating the identity and density of the dopants in a crystal matrix, and by combining more than one dopant ion in the host matrix, a variety of emissions from the NIR to the UV can be realized to tune the optical properties of these solid-state materials. Currently, the most common trivalent lanthanide emitters, or “activators”, used in UCNP systems are Tm^3+^, Er^3+^, and Ho^3+^. The emissions from these ions are efficiently “sensitized” by energy transfer from Yb^3+^, permitting multicolor emissions at a single excitation wavelength of 980 nm [1]. The emission spectrum of Tm^3+^ consists of a band at 800 nm, originating from the ^3^H_4_ → ^3^H_6_ transition, with two other higher energy bands in the violet and blue region with wavelengths 452 nm and 476 nm resulting from the ^1^D_2_ → ^3^F_4_ and ^1^G_4_ → ^3^H_6_ electronic transitions, respectively. The emission spectrum of Er^3+^ shows three distinct bands, two of which are centered in the green region of the visible spectrum at 525 and 545 nm, originating from the ^2^H_11/2_ → ^4^I_15/2_ and ^4^S_3/2_ → ^4^I_15/2_ electronic transitions, respectively, and one in the red region centered at 660 nm resulting from the ^4^F_9/2_ → ^4^I_15/2_ transition. For Ho^3+^, two main emissions bands are observed, one of which is green at 541 nm and one red at 647 nm, arising from the ^5^S_2_/^5^F_4_ → ^5^I_8_ and ^5^F_5_ → ^5^I_8_ electronic transitions, respectively [3]. Many strategies for optical tuning have been reported, and a classical effective approach was first disclosed by Haase in which NaYF_4_ crystals were doped with 20% Yb^3+^, 2% Er^3+^ and 20% Yb^3+^, 2% Tm^3+^ yielding yellow (a combination of green and red emissions) and blue emissions, respectively [4]. Figure 1 presents an image that has become popular in the field of UCNPs that describes the origin of multicolor emissions through manipulation of dopant combinations. Table 1 provides an overview of some of the major emission wavelengths that can be generated by lanthanide doped UCNPs [4–19]. Table 1 is not intended to be comprehensive and rather provides perspective of how emissions can be tuned to facilitate matching to detector technologies and the specific criteria associated with the development of assays.

In UCNP systems, the concentration of sensitizer Yb^3+^ ions is often kept quite high (∼20 mol%) to ensure efficient absorption of incident excitation radiation and subsequent energy transfer, while the concentrations of activator ions are generally kept low (∼0.2 mol%–5 mol%) to eliminate deleterious cross-relaxation effects that can happen through ion-ion interactions within the host lattice. These deleterious interactions, however, can be rationally exploited to tune the optical properties of the material by intentionally quenching emissions from a particular energy transition while concurrently promoting another. Such an optical tuning strategy based on cross-relaxation processes was first reported by Chen *et al.*, where they doped cubic (α) phase NaYF_4_: Yb^3+^, Ho^3+^ UCNPs with Ce^3+^ to change the photoluminescence (PL) output of the material from green (541 nm) to red (647 nm) [14]. It was proposed that the addition of Ce^3+^ into the host lattice promoted cross-relaxation of populations in the green emitting state to the red emitting state through the following two resonant cross-relaxation processes: ^5^S_2_/^5^F_4_ (Ho^3+^) + ^2^F_5/2_ (Ce^3+^) → ^5^F_5_ (Ho^3+^) + ^2^F_7/2_ (Ce^3+^) and ^5^I_6_ (Ho^3+^) + ^2^F_5/2_ (Ce^3+^) → ^5^I_7_ (Ho^3+^) + ^2^F_7/2_ (Ce^3+^). Zhang and co-workers have also exploited this strategy to tune the relative emission intensities of NaYF_4_: Yb^3+^, Tm^3+^ UCNPs, which exhibit violet (452 nm) and blue (476 nm) emissions [9]. By increasing the concentration of Tm^3+^ in the host lattice, the effective ion-to-ion distance is decreased. This results in enhancement of cross-relaxation between adjacent Tm^3+^ ions, providing an increase in the population of violet emitting states and a decrease in blue emitting states. The ability to manipulate green and red emissions from β-NaYF_4_: Yb^3+^, Er^3+^ UCNPs by doping with Mn^2+^ has also been demonstrated [15]. The mechanism responsible for the observed red shift was explained in terms of a cross-relaxation phenomenon whereby Mn^2+^ ions in the host lattice quench radiative pathways leading to green emissions while concurrently promoting those leading to red emissions. Alkali doping strategies have been extensively explored as a strategy to tune the structure and emissions profiles of Ln^3+^ doped UCNPs, and this topic has recently been reviewed [20]. Table 1 includes some representative examples of the wavelengths that can be generated by a variety of UCNPs that exploit cross-relaxation phenomenon to tune UC emissions profiles.

While it is possible to exploit the process of cross-relaxation judiciously to tune the emissions of UCNPs, for some applications it is desirable to eliminate the cross-relaxation phenomenon and minimize surface quenching effects to increase the overall luminescence efficiency of the material through spatial isolation of activator ions. This can be achieved by means of a core-shell architecture [3]. Core-shell nanoparticle constructs are fabricated by epitaxial growth of inorganic nanocrystals, as recently reviewed [1]. If the conditions for epitaxial growth are not met, the formation of “hybrid” or “nanocomposite” structures can be realized, which in themselves exhibit unique optical properties. In most early reports, the shell was inert and composed of the same material as the host, serving solely to enhance the luminescence intensity of the UCNP by blocking surface quenching sites. Qian and Zhang were the first to report a core-shell UCNP where both the core and shell were doped with activator Ln^3+^ ions [16]. NaYF_4_: Yb^3+^, Tm^3+^ cores were coated with a uniform NaYF_4_: Yb^3+^, Er^3+^ shell, yielding materials that exhibited multiple emission peaks at 450, 475, 409, 520, 541, and 653 nm arising from the ^1^D_2_ → ^3^F_4_ and ^1^G_4_ → ^3^H_4_ electronic transitions of Tm^3+^, and the ^4^H_9/2_, ^4^H_11/2_, ^4^S_3/2_, and ^4^F_9/2_ → ^4^I_15/2_ transitions of Er^3+^, respectively. This strategy was extended in the preparation of core-shell-shell NaYF_4_: Yb^3+^, Tm^3+^/NaYF_4_: Yb^3+^, Er^3+^/NaYF_4_: Yb^3+^, Tm^3+^ UCNPs exhibiting tunable emissions extending from the NIR to UV spectral regions after excitation using 980 nm radiation [10]. The addition of an outer shell structure doped with Tm^3+^ enhances the overall emission of Tm^3+^, and offers a route to selectively control the ratio of intensity from the various emission bands. Core-shell architectures have also been implemented in the fabrication of UCNPs doped with so-called “non-conventional” sensitizer and activator Ln^3+^ ions, broadening the scope of absorbance and emission spectra that can be produced by such materials [17]. NaGdF_4_: Yb^3+^, Tm^3+^ core UCNPs coated with a NaGdF_4_:Ln^3+^ (Ln^3+^ = Tb^3+^, Dy^3+^, Sm^3+^ or Eu^3+^) shell show multiple emission peaks spanning the visible spectrum (summarized in Table 1).

Other versions of shell structure have been developed for UCNPs. For example, shells made of amorphous silica have been used to tune the emissions of UCNPs by embedding in them conventional down converting (DC) materials. These include organic dyes or quantum dots (QDs), which accept gathered excitation energy from the upconverting core via luminescence resonance energy transfer (LRET). The addition of such down converting (DC) materials in the shell allows for substantial flexibility in the selection of emission wavelengths of UCNPs [8]. NIR UC materials coated with multiple QD layers have also been shown to emit light throughout the entire visible spectrum following 1064 nm excitation [21]. Analogous LRET processes have also be used to selectively quench UCNP emissions bands providing tunable UC emissions using QDs [19] as well as gold nanoparticles (AuNPs) [22]. Some representative examples of core-shell tuning strategies and emission wavelengths are included in Table 1.

Optical tuning of UCNP materials is readily achieved and has been described in a number of review articles [1,3,23]. Despite the control that has been demonstrated, the quantum efficiency of these materials is typically quite low (less than 1%). For practical applications of UCNPs in integrated detector technologies, emission enhancement strategies are desirable to increase the observed luminescence intensity. This can be paramount in achieving a high level of sensitivity for development of assays. One strategy for achieving emission enhancement is to bring the UC material into close proximity with a metallic surface, either by coating the material with a gold or silver shell or by association with metallic nanostructures at distances where electrical fields can overlap. The signal enhancement that is realized is typically attributed to coupling with a localized surface plasmon resonance (LSPR) phenomenon, whereby the collective oscillation of electrons at the surface of the metal upon interaction with incident light of a specific wavelength acts to concentrate the electric field around the particle. LSPR has been shown to enhance the fluorescence of different emitters such as QDs and organic dyes, often by many orders of magnitude, when the distance between the metal surface and the emitters are optimized [24,25].

In an early study, Feng and coworkers reported a 2.3 and 3.7 fold enhancement in green and red emission, respectively, when UCNPs with dual emission peaks were deposited on Ag nanowires [26]. This was soon followed by work that investigated controlled coupling of UCNPs to metallic surfaces [25] in which Au nanospheres were moved in a well-defined fashion towards the UCNP by atomic force microscopy (AFM) in contact mode. As anticipated, the enhancement that was observed depended on the distance between the NPs as well as the polarization of the excitation radiation. Moreover, a reduction in both rise and decay times was reported. The reduction in rise time was attributed to a synergic effect of the increased excitation intensity and the coupling of 540 nm plasmon resonance from the AuNP. The reduction in fluorescence lifetime was attributed from the quenching effect often observed with metal NPs, which promoted nonradiative pathways. Numerous publications have since described the exploration of different approaches for the coupling of UCNPs with metal NPs, including the growth of Au NPs or Au nanoshells [27–29] directly onto UCNPs, adsorption of Au NPs to UCNPs [30], separation by metal NP and UCNPs by atomic layer deposition of Al_2_O_3_ [24], and 3D plasmonic nano-antennas [31]. Different extents of enhancements have been observed, with the 3D plasmonic nano-antennas giving the largest enhancement of 310 times that of the UCNP alone.

### 2.3. UCNPs in Bioassays

Since IR excitation allows for high penetration depth into biological tissues, minimizes damage to living organisms, and ameliorates autofluorescence and scatter, the excitation of UCNPs in the near NIR makes them very attractive for *in vivo* applications as imaging agents [1,32,33]. Due to properties such as optical tuning and narrow emission bands, UCNPs are also of interest to applications in bioassays and have been used as both passive labels and LRET donors for the detection of a variety of small molecules and biological compounds.

UCNPs were first used as reporters to detect cell antigens in 1999 [34] and this work demonstrated the reduction in autofluorescence that was possible with NIR excitation. UCNPs have been used in a variety of assay methodologies. An immunochromatographic assay format was described by Hampl *et al.* [35] for the detection of human chorionic gonadotropin (hCG) using UCNPs as reporters. This method achieved a detection limit of 1 pg and a dynamic range spanning over three orders of magnitude. A lateral flow assay format has been described for the detection of bacteria and small molecules [36]. Using upconverting particles (UCPs) as labels, 10^3^ org·mL^−1^ *Escherichia coli* (*E. coli*) bacteria were detected in a background of 10^9^ org·mL^−1^ culture medium. In contrast to the use of a sensitive Au NP based colorimetric method, only tens to hundreds of UCPs were required to generate a detectable signal whereas tens of thousands of Au NPs are needed to achieve equivalent sensitivity. UCNPs have been used for the detection of various other bacterial species, such as *M. tuberculosis*, *S. pneumonia* and *Y. pestis* [37,38].

**Figure 2.**
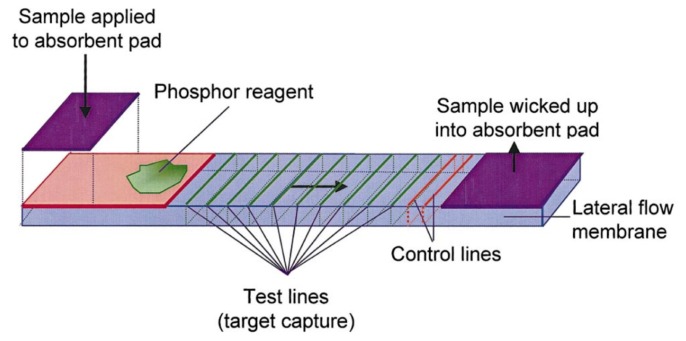
Schematics showing the operation of the lateral flow test strip [36].

UCNPs have been used in assays for the detection of other targets, such as metal ions, toxins and metabolites [39–41]. For example, Niedbala and coworkers have demonstrated the potential of using UCNPs for the detection of different drugs of abuse, such as amphetamine, methamphetamine, phencyclidine and opiates in saliva, with 1 ng·mL^−1^ limit of detection [36]. In this latter study, a sandwich assay format using antibodies as biorecognition elements was used on a lateral flow test strip. The primary antibody was immobilized on the surface and the analyte was transduced using a secondary antibody labelled with UCNP. Test lines differentially provided optical signal for either the target or control, and signal intensities from test lines could be used to quantify the concentration of target in a sample, as depicted in Figure 2.

In another study for the detection of antigens, multiple targets were detected simultaneously using UCNPs with different emission profiles [42]. Aflatoxin B_1_ (AFB) and ochratoxin A (OTA), two types of mycotoxins, were simultaneously quantified in a competitive assay format. Using Tm^3+^ and Er^3+^ doped UCNPs decorated with anti-AFB and anti-OTA antibodies, respectively, the emission band at 452 and 660 nm were used to quantitatively identify the targets.

A further example is a system using LRET as a transduction scheme for the detection of glucose in solution [43]. In this study, UCNPs were used as the LRET donor and rhodamine B isothiocyanate (RBI) conjugated to 3-aminophenylboronic acid (APBA) was used as the acceptor. Glucose molecules were immobilized onto UCNPs. The APBA component of the RBI-APBA conjugate was then allowed to bind the glucose on the surface of UCNP, bringing the RBI into close proximity of the UCNP so that LRET would occur. When a sample solution containing glucose was added, the free glucose in solution competed for the APBA, and the RBI-APBA complex dissociated from the UCNP-glucose hindering LRET. The decrease in acceptor luminescence was used to determine the concentration of glucose in solution.

**Figure 3.**
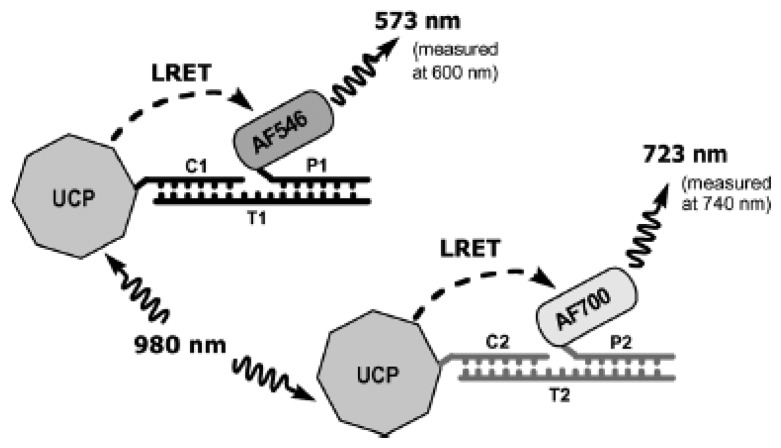
Schematics of UCNP based LRET system. The upconverting particle (UCP) is excited using 980 nm laser and serves as energy donor to the dye-labelled reporters (P1 and P2). The LRET sensitized emissions from the dyes are used for detection [48].

UCNPs have also been implemented extensively for DNA detection. They have been used both as direct labels and in energy transfer applications in a variety of formats, including hybridization assays and sandwich assays, both in solution and on solid supports [44–47]. One interesting example is the use of UCNP in a single donor, multiple acceptor LRET system [48]. In this study, one type of UCNP with multiple emission peaks (540 nm and 653 nm) was used as the donor for two different organic dyes, AF546 and AF700. After the UCNPs were covalently modified with the two types of capture DNA sequences, the target strands were first incubate with the dye-labeled reporter DNA before introducing the UCNP-capture DNA. After incubation, the UCNPs were excited with NIR radiation at 980 nm and emissions of the dyes were measured at 600 nm and 740 nm (Figure 3).

### 2.4. Time Resolved Measurements

**Figure 4.**
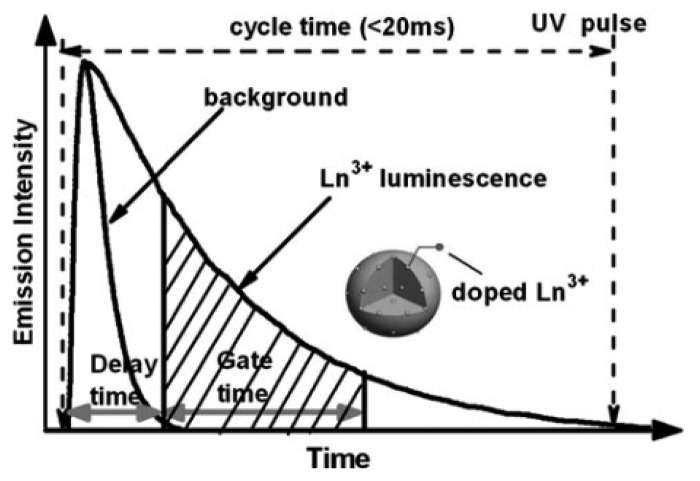
The principle of the time resolved technique. The background signal decays during a delay time and luminescence from the Ln^3+^ doped particle is collected in the gate time [52].

The sensitivity and detection limit of optical bioassays that rely on emission of radiation can be limited by autofluorescence and scatter. The use of long-wavelength excitation in combination with UCNPs for bioassays has the potential to increase signal-to-background ratio and improve detection limits. The UC process associated with excitation using near-IR and IR radiation typically suffers from a low quantum yield, often being below 3% [1]. However, when such lanthanide-doped nanoparticles are excited in the UV region of the spectrum, the down conversion processes can exhibit quantum yields as high as 68% [49]. To eliminate the background signal associated with UV excitation, a pulsed excitation source is used and the emissions from lanthanides, which have lifetimes on the order of milli-seconds, are measured after the short lived background from autofluorescence and scatter has decayed in nanoseconds. The principle of time resolved luminescence (TRL) measurements is outlined in Figure 4. The use of lanthanide doped NPs in TRL has only recently been reported. However, the use of lanthanide chelates in time-resolved bioassays dates back to the mid-seventies. Lanthanide chelates have been reported for various applications such as the detection of biomolecules, visualization of live cells and analyses of metabolites [50,51].

The sensitivity of assays based on lanthanide chelates has been improved by incorporation of the chelates inside nanoparticles to increase the lanthanide-to-biomolecule ratio. For example, over 30,000 Eu(III)-2-thenoyltrifluoroacetone complexes were incorporated in 107 nm polystyrene beads, giving a 4 orders of magnitude dynamic range for the detection of PSA in levels as low as 250 zeptomoles [53]. This represents a 100-fold improvement in assay sensitivity compared to a conventional Eu-labeled streptavidin assay. However the application of such particles is limited due to their inherent large size and swelling, in addition to the leakage of the lanthanide complexes [50]. Lanthanide chelates in silica NPs represent an alternative structure and have been used in time resolved fluoroimmunoassays and time resolved luminescence imaging applications [54–56]. The use of lanthanide doped NPs such as NaYF_4_ [57], KGdF_4_ [58], CaF_2_ [59], ZrO_2_ [60], LaPO_4_ [49] and SiO_2_ [61] have been reported to offer better photostability than chelates due to the rigid crystal lattice. Lifetimes exhibited by such nanoparticles are summarized in Table 2.

The use of lanthanide doped GdF_3_ nanoparticles as passive labels for time resolved detection of trace amounts of avidin was reported to provide a limit of detection of 74 pM [52]. Chen *et al.* have reported the use of lanthanide-doped NPs in combination with UV excitation and time resolved luminescence resonance energy transfer (TR-LRET) using organic dyes as acceptors. The apparent lifetime of organic dye acceptors is increased since the long lived excited states of Ln^3+^ slowly populate the excited state of acceptor. This allows for the LRET signal to be free of interferences from short-lived background signals [57]. Chen's group demonstrated the first TR-LRET based assays for the detection of avidin employing biotin labeled NaYF_4_:Ce/Tb UCNPs and fluorescein isothiocyanate (FITC)-labeled avidin as acceptor, and reported a dynamic range spanning 4.8 nM–400 nM. The NPs were excited at 290 nm and exhibited significant emission centered at 489, 542, 585 and 623 nm. PL lifetimes based on 542 nm peak was determined to be 2.21 ms for biotinylated NPs and decreased to 0.7 ms with increasing acceptor concentration. In addition to eliminating background signals, TRL allowed for distinction between direct excitation of FITC and LRET sensitized emission based on apparent lifetimes. A similar UC-LRET methodology using Y_2_O_2_S:Er/Yb upconverting phosphors and fluorescent phycobiliprotein as acceptor demonstrated similar dynamic range 0.7–9.0 nM [62]. Other examples of TR-LRET have been reported in the literature [58–60].

Most lanthanide-based TRL assays are reported in microtitre plates and have made use of commercially available plate readers with TRL capability. Examples include implementation of SpectraMax Paradigm instruments from Molecular Devices and the Infinite m1000 system from Tecan. Time-delayed detection in plate readers is typically based on oscillator-controlled electronics. A flash lamp, laser or LED or a combination of the above is used as an excitation source and detection is facilitated by filters or monochromators. In many cases, photo multiplier tubes (PMTs) with exceptionally low dark count rates are needed for nanoparticles exhibiting low quantum yields [50].

**Figure 5.**
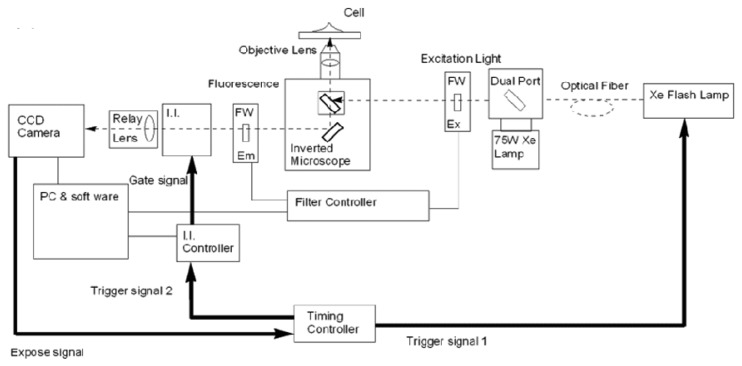
Schematic diagram of the TRLLM system. The excitation light from a xenon flash lamp passes through an excitation filter and is focused onto cells with dichroic mirrors. The emitted radiation travels through an emission filter, after which the image intensifier unit (I.I.) passes the long-lived luminescence to the CCD camera [69].

Microscopy systems for time-resolved lanthanide measurements have been reported and are often based on mechanical choppers [63–66] and can make use of image intensifier units [67,68] as a basis for achievement of time resolution. Implementation of an image intensifier facilitates control of gate and delay times and achieves better uniformity of light at the detector. Hanaoka *et al.* reported a time-resolved, long-lived luminescence microscopy (TRLLM) imaging system for the detection and visualization of biomolecules in live cells [69]. The schematic in Figure 5 shows the use of a xenon flash lamp as an excitation source, a time controller and an image intensifier unit. The TRLLM system was used for imaging lanthanide complexes that served as biolabels, in addition to monitoring of intracellular Zn^2+^ using a luminescent lanthanide-based probe.

## 3. CMOS Microsystem

The capability of integrating a large array of detectors with signal processing circuits, low power consumption, and low fabrication cost in high volume production are well-known features offered by the CMOS technology. In this section, three specific attributes of CMOS particularly beneficial to the integration with luminescence bioassays are examined, namely system miniaturization using contact imaging, spectral detection, and time-resolved detection.

### 3.1. System Miniaturization

In a conventional excitation-induced luminescence imaging system, the signal path from the excitation source to the detector often consists of bulky and expensive optical components such as a system of lenses. Contact imaging, in contrast, is a recent technique whereby the object to be imaged is placed near or directly on top of the photodetector array [70–72]. It does not require intermediary optics such as lenses, resulting in orders of magnitude improvement in sensitivity as well as significant size and cost reduction [73]. These advantages make contact imaging attractive for portable low-cost biosensor applications.

The choices of the photodetector for conventional luminescence imaging systems have been the PMT and the charge-coupled device (CCD). PMTs are amongst the most sensitive photodetectors, but are bulky, expensive and require a high operating voltage, making them unattractive to be integrated into a miniaturized system. The throughput of PMT-based detection systems is relatively low due to the lack of parallelism. CCDs can be employed in an arrayed implementation, but do not allow for the on-silicon-chip integration of peripheral circuits such as signal conditioning circuits. This increases cost and limits miniaturization. CMOS technology, on the other hand, has the advantages of low cost, high integration density, and signal processing versatility. Numerous recent designs based on the CMOS p-n-junction photodiode have been reported for many bioluminescence detection lab-on-chip devices [74–78]. The monochromatic photogate structure typically used in a CCD has been demonstrated in CMOS. However, the exploitation of the CMOS polysilicon photogate to perform spectral sensing has largely been unexplored.

To illustrate key concepts of miniaturization by combining contact imaging and CMOS spectral detection, key design concepts of a CMOS fluorescence analysis microsystem [79] is discussed. The microsystem integrates a high-performance optical interference filter and a 128 × 128 pixel active pixel sensor fabricated in a standard 0.35 μm CMOS technology. The thin-film filter has an optical density greater than 6.0 at the wavelength of interest, providing ample excitation rejection to the 532 nm solid-state laser. Microsystem performance is experimentally validated by imaging spots of the Cyanine-3 fluorophore, conventionally used in DNA detection. Detection limit was measured to be 5000 fluorophores/μm^2^.

**Figure 6.**
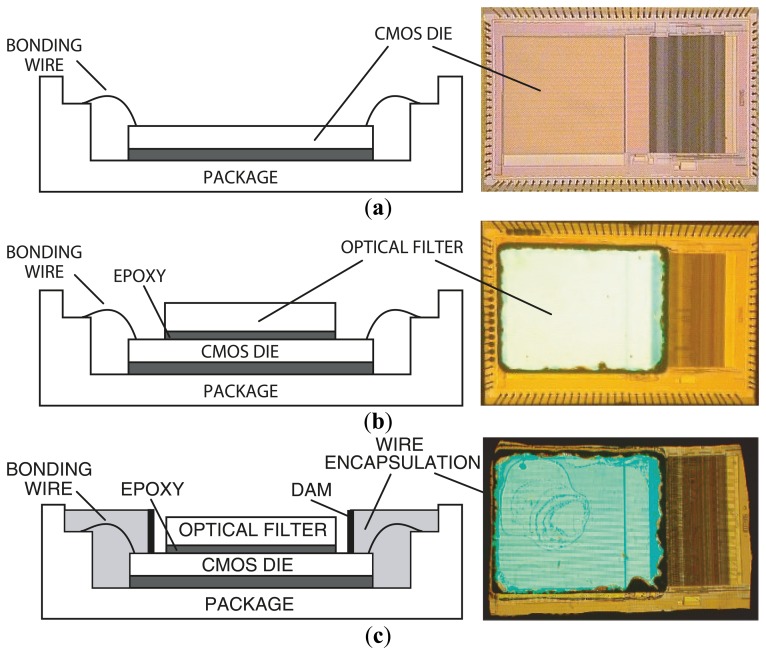
(Left) cross-sectional diagram and (right) die micrograph for three key microsystem integration steps. (**a**) CMOS die is packaged and wire bonded. (**b**) Optical filter is attached to the CMOS die using a refractive-index-matched epoxy. (**c**) Bonding wires are encapsulated with an epoxy for protection [79].

Figure 6 describes the steps in microsystem integration. Figure 6a shows the CMOS die bonded and packaged using a standard wire bonding and packaging process. The CMOS die was raised inside the chip package by placing a die spacer below it. This brings the CMOS photo detector array closer to the top surface of the chip package, where the sample is placed. Figure 6b shows the attachment of the optical filter to the CMOS die using a refractive-index-matched epoxy, which eliminates unwanted light reflections at the filter–CMOS die surface. Finally, as depicted in Figure 6c, an opaque epoxy dam is built around the optical filter. Then, an epoxy is poured over the bonding wires. This bonding wire encapsulation electrically insulates the bonding wires, protects them, and prevents stray reflected laser light from leaking through the sides of the filter thus reaching the sensor array.

The optical filter chosen for the microsystem is a discrete thin-film interference filter, which was designed, fabricated (Omega Optical), and optically tested prior to integration with the CMOS die. This also makes the system more flexible in serving a wide variety of applications. To adapt the system for UCNP bioassays, the 570 nm long pass filter can be readily replaced with a 900 nm short pass filter to suit the NIR excitation of UCNPs. An alternative to post-fabrication filter attachment is direct deposition. However, direct thin-film deposition over the CMOS die leads to higher complexity due to complications in adjusting the fabrication process to compensate for the temperature and material differences between the surface of the optical filter and the CMOS die. The masking of bonding pads during the coating process in the direct deposition method also adds additional complexity. In the presented approach, a 100 μm-thick 25 mm × 25 mm optical filter is diced into smaller pieces of size 2.2 mm × 2.8 mm each (by diamond saw, performed at Corwil). The small-sized filter is then attached to the CMOS die. The interference filter was fabricated using 60 layers of Nb_2_O_5_ and SiO_2_. The coatings were deposited onto a 100 μm-thick microsheet of fused silica substrate by physical vapor deposition.

### 3.2. CMOS Spectral Detection

**Figure 7.**
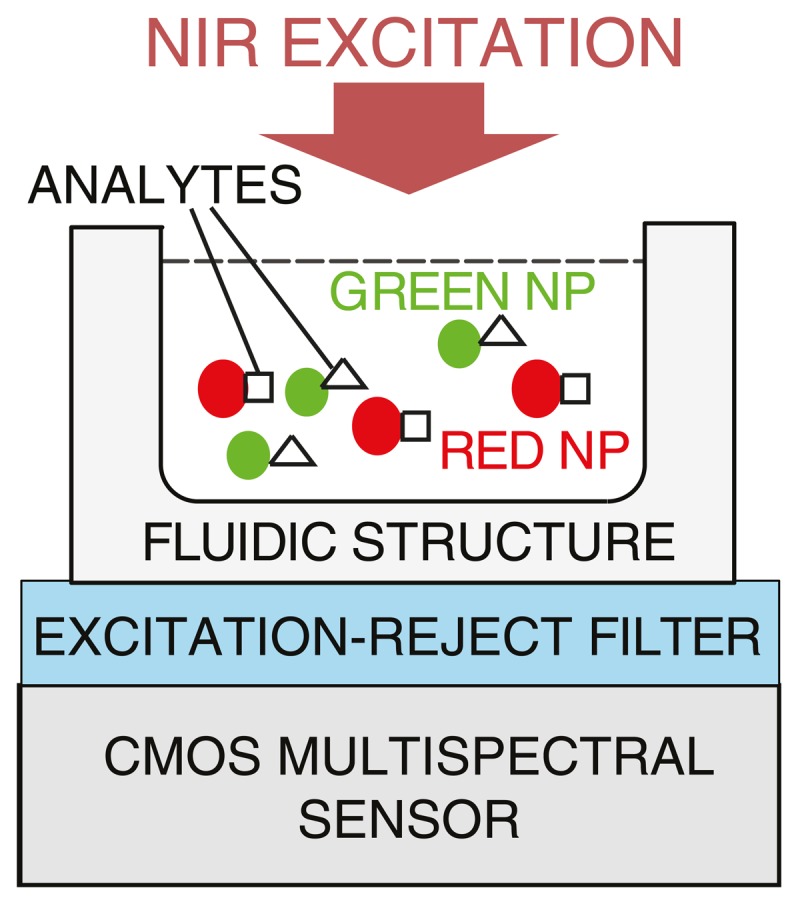
Schematic of a CMOS luminescence detection microsystem (NP = nanoparticle).

Optical tuning of emission wavelengths for UCNPs enables unprecedented flexibility in spectral multiplexing. For example in DNA detection, different DNA target sequences can be tagged with labels that emit light at different wavelengths, which can be concurrently detected and distinguished. Unlike other spectroscopic techniques, such as Raman spectroscopy, where continuous fine spectral resolution is required, luminescence imaging requires spectral differentiation among only a few discrete wavelengths, typically well-separated (*i.e.*, <50 nm apart) in the visible band [70]. To avoid the complexity and variability associated with surface chemistry common to spatially multiplexed arrays such as microarrays, spectrally-multiplexed assays using UCNPs can be developed, as depicted in Figure 7.

**Figure 8.**
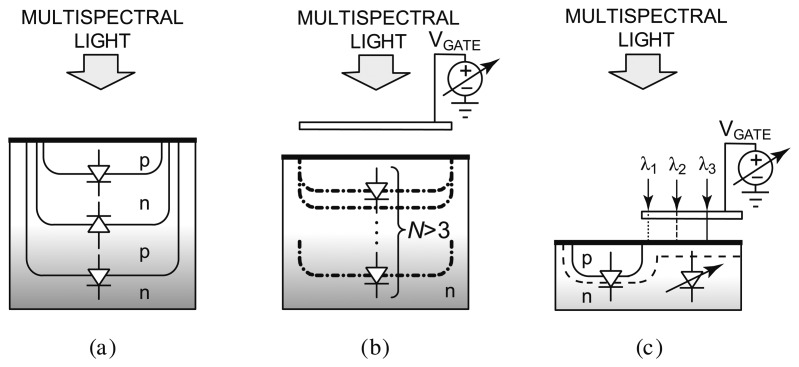
Evolution of CMOS filterless spectral sensing approaches. V_GATE_ is a control voltage. (**a**) Buried triple p-n-junction embedding diodes at three fixed depths [82], (**b**) photo sensing region depth modulation enabling light collection at multiple electronically-tunable depths in custom CMOS [83], and (**c**) CMOS color photogate (CPG) consisting two sensing regions, one of which is tunable by the control voltage and covered by a poly-Si gate.

Recently, CMOS technology has been researched for integrated spectral detection. Requiring a reduced amount of external optical components or none at all, these CMOS systems enable a new breed of compact, low-cost luminescence detection systems. Figure 8 illustrates the evolution of CMOS filterless spectral sensing approaches. Conventionally, differentiation between luminescence emission wavelengths has been achieved by using a set of optical bandpass filters to select different parts of the emission spectrum. The optics involved is bulky and expensive. To circumvent this problem, other spectral methods have also been investigated. Methods based on diffraction grating (the splitting of light) [80] and Fabry-Perot etalon (tuned resonance cavity) [81] generally offer high spectral resolution, but require micromachining and post-processing such as wafer polishing and wafer bonding. Eliminating the need for sophisticated optics and post-processing is the ultimate remedy to the high design complexity and fabrication cost.

Techniques that solely rely on integrated circuit process technology have been developed, most notably buried junction technology [82,84–86], on which the Foveon sensor [87] is based, as shown in Figure 8a. Since light absorption in a semiconductor varies across wavelengths in such a way that light of a longer wavelength can penetrate deeper, a photocurrent measured at a deeper depth consists of stronger long wavelength components. By sensing at several depths, color information can be inferred. Although the buried junction approach achieves high spatial density and is suitable for photographic applications requiring only three colors (e.g., blue, green, and red), there is a limit to the number of diodes that can be implemented into a vertical stack, for example three for a dual-well process. This renders it unsuitable for applications that require sensing more than three wavelengths. To overcome this limitation, a spectrally-sensitive photodiode that can potentially sense more than three colors has been developed [83], as shown in Figure 8b. A biased poly-silicon gate modulates the photo sensing region depth to effectively achieve an equivalence of many buried p-n-junctions. However, the reliance on the vertical dimensions of the CMOS process technology limits the scalability of the device dimensions. The most recently reported prototype is fabricated in a 5 μm custom process [88].

**Figure 9.**
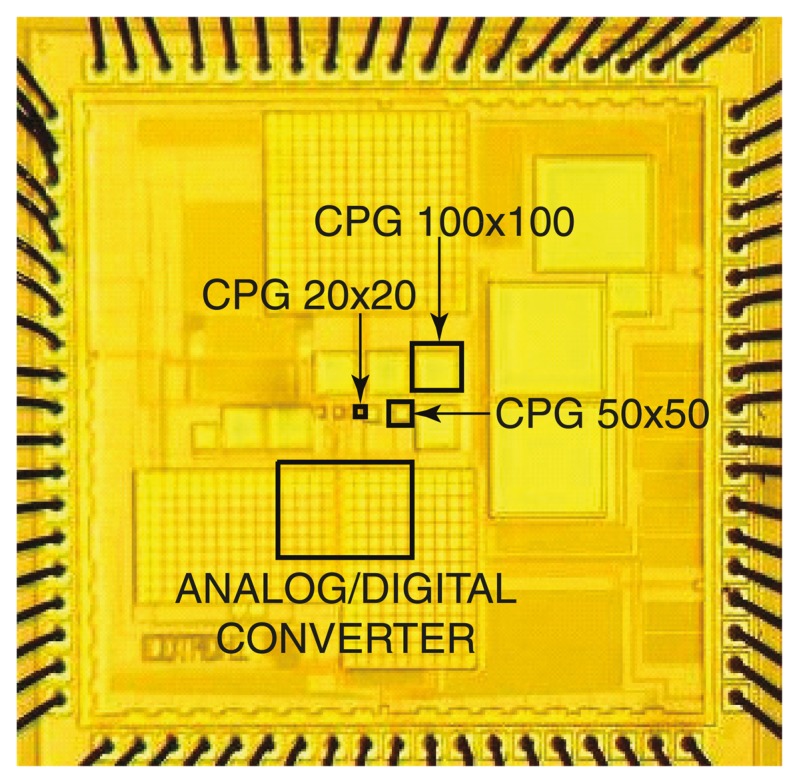
Die micrograph of a 2 mm × 2 mm sensor test chip fabricated in 0.35 μm standard CMOS technology. Depicted are three differently sized CMOS photogate (CPG) test structures with an on-chip analog-to-digital converter.

Figure 8c depicts a new approach that employs a voltage to tune the spectral response of a detector [89–91]. Sensing of a small set of well separated wavelengths (e.g., >50 nm apart) is achieved by tuning the spectral response of the device with a bias voltage. Termed the CMOS photogate (CPG), it employs the polysilicon gate as an optical filter, which eliminates the need for an external color filter. Multiple measurements with different color components are collected. A back-end algorithm then solves the inverse problem by computing the different color intensity components from prior knowledge of the dependency of detector response to voltage. A prototype has been fabricated in a standard 0.35 μm digital CMOS technology, depicted in Figure 9. It demonstrates intensity measurements of blue (450 nm), green (520 nm), and red (620 nm) illumination with a peak signal-to-noise ratio (SNR) of 34.7 dB, 29.2 dB, and 34.8 dB, respectively. The prototype has been applied to detect green-emitting quantum dots (gQDs) and red-emitting quantum dots (rQDs). It spectrally differentiated among multiple emission bands, effectively implementing on-chip emission filtering. The prototype demonstrated single-color measurements of gQD and rQD concentrations to a detection limit of 24 nM, and multi-color measurements of solutions containing both colors of QDs to a detection limit of 90 nM and 120 nM of gQD and rQD, respectively.

To illustrate the application of spectral multiplexing in DNA analysis, a fluorescence contact imaging microsystem [92] is described below. The microsystem integrates a filterless CMOS CPG sensor that exploits the polysilicon gate as an optical filter. The CPG is applied to fluorescence-based transduction in a spectrally multiplexed format by differentiating among multiple emission bands, hence replacing the functionality of a bank of emission filters. The CPG was fabricated in a standard 0.35 μm CMOS technology. The multi-color imaging capability of the microsystem in analyzing DNA targets has been validated in the detection of marker gene sequences for the spinal muscular atropy disease and the *E. coli* bacteria. Spectral-multiplexing enabled the two DNA targets to be simultaneously detected with a measured detection limit of 240 nM and 210 nM for the two target concentrations at a sample volume of 10 μL for the green and red transduction channels, respectively.

### 3.3. CMOS Time-Resolved Measurement

Similar to conventional fluorophores or quantum dots, UCNPs can also be used in time-resolved measurements. Time-resolved imaging is a technique where low-light signals are recorded with high timing resolution relative to a synchronized optical impulse excitation, in order to extract the characteristic luminescence decay constant or lifetime [93]. For example in time-correlated single photon counting (TCSPC), a typical apparatus includes a pulsed optical source, a discrete detector such as an avalanche photodiode (APD) or PMT, external time-to-digital converter (TDC) and a workstation to compute the decay constant, resulting in a bulky, expensive and power-hungry acquisition system. A major limitation of this approach is the restrictively low photon count limit that is below 5% of the excitation rate, which is necessary to avoid distortion due to photon pile-up caused by both long detector dead-time and the inability of the TDC to process more than one event per excitation period. Therefore, TCSPC has in the past been limited by peak acquisition rates of around 1 MHz [94].

Recent advances in single-photon avalanche diodes (SPADs) and on-chip TDCs implemented in standard CMOS processes have enabled TCSPC measurements to be performed by an imaging array, and with much higher time resolution [94]. To assess the state-of-the-art in CMOS integrated systems for time-resolved imaging, several recently reported prototypes with their features and implementation approaches are summarized next.

A CMOS image sensor with direct digital phase output is reported in [95]. A row-level zero-crossing detection is implemented to extract the phase-shift between the intensity modulated excitation signal and the emitted fluorescence, generating a time delay signal proportional to the fluorescence lifetime of the target analyte. A time-interpolated TDC is subsequently used to quantize the time delay into a digital representation of the phase-shift for post-signal processing and image reconstruction. The design has been implemented in a low-power 65 nm CMOS technology. The TDC features a temporal resolution of 110 ps over a 414 s range, which corresponds to a dynamic range of 132 dB. Extensive characterization results demonstrate a phase readout sensitivity of better than 0.01 degrees at a 1.2 kHz modulation frequency and 0.1 degrees at up to 1 MHz. This design is an example of the high-precision time-domain processing capability CMOS technology can achieve.

Much engineering has gone into improving the optical signal handling capability of the CMOS technologies. In [96], a time-resolved CMOS image sensor with draining-only modulation (DOM) pixels for time-domain fluorescence lifetime imaging is reported. In the DOM pixel, which uses the pinned photodiode (PPD) technology, a time-windowed signal charge transfer from a PPD to a pinned storage diode (PSD) is controlled by a draining gate only, without a transfer gate between the two diodes. This structure allows potential barrierless and trapless charge transfer from the PPD to the PSD. A 256 × 256 pixel time-resolved CMOS imager with 7.5 × 7.5 μm^2^ DOM pixels has been implemented using 0.18 μm CMOS image sensor process technology with PPD option. The prototype demonstrated high sensitivity for weak signal of less than one electron per light pulse, and accurate measurement of fluorescence decay with subnanosecond time resolution.

A CMOS imager with column-level 10-bit TDC array for time-resolved optical sensing is reported in [97]. This imager is one of the first fully integrated system for photon time-of-arrival evaluation. The sensor comprises an array of 128 × 128 single-photon pixels, a bank of 32 TDCs, and a 7.68 Gbps readout system. Outstanding timing precision of single-photon avalanche diodes and the optimized measurement circuitry enable a resolution of 97 ps within a range of 100 ns. Accurate measurements are achieved based on a short integration time of 50 ms even when signal photon count rates as low as a few hundred photons per second. Aside from fast fluorescence lifetime imaging, the system is also suitable for applications include 3-D imaging (*i.e.*, distance measurement), optical range finding and, more generally, imaging based on time-correlated single photon counting.

A CMOS image sensor with in-pixel two-stage charge transfer for time-resolved fluorescence lifetime imaging with sub-nanosecond time resolution is presented in [98]. In order to analyze the fluorescence lifetime, the proposed CMOS image sensor has two charge transfer stages using a pinned photodiode structure in which the first charge transfer stage is for the time-resolved sifting of fluorescence in all the pixels simultaneously and the second charge transfer stage is for reading the signals in each pixel sequentially with correlated double sampling operation. A 0.18 μm CMOS image sensor technology with a pinned photodiode process option is used for the implementation of a 256 × 256 pixel CMOS image sensor. The decaying images and lifetimes of fura-2 solutions having different concentrations have been measured with a 250 ps time step using an ultraviolet laser diode as a light source.

A fully digital single-chip solution to fluorescence lifetime sensing implementing lifetime estimation on-chip using the centre-of-mass method (CMM) is presented in [94]. The device comprises a 1.3 × 1.7 mm^2^ CMOS chip in 0.13 μm technology, featuring an array of SPAD detectors in a silicon photomultiplier architecture, the multichannel TDC architecture, a CMM processing block and a serial interface for control and data capture. This design provides mitigation to the pulse pile-up problem. A time-multiplexed, multichannel TDC architecture is introduced to allow the pile-up limit to be broken, achieving a maximum 100 Mphoton/s acquisition rate, allowing lifetime decays of common organic fluorophores to be obtained.

A miniaturized, high-throughput, time-resolved fluorescence lifetime sensor implemented in a 0.13 μm CMOS process is described in [99]. The design combines single photon detection, multiple channel timing and embedded pre-processing of fluorescence lifetime estimations on a single device. Detection is achieved using an array of SPADs arranged in a digital silicon photomultiplier architecture with 400 ps output pulses and a 10% fill-factor. An array of TDCs with 50 ps resolution records up to 8 photon events during each excitation period. Data from the TDC array is then processed using the CMM method to produce fluorescence lifetime estimations in real-time. The system was demonstrated in a practical laboratory environment with measurements of a variety of fluorescent dyes with different single exponential lifetimes, showing the ability of the sensor to overcome the classic pile-up limitation of TCSPC by over an order of magnitude.

A two-chip micro-scale time-resolved fluorescence analyzer integrating excitation, detection, and filtering is described in [100]. This design achieves a new level of system integration. A new 8 × 8 array of drivers realized in standard low-voltage 0.35 μm CMOS is bump-bonded to AlInGaN blue micro-pixellated light-emitting diodes (micro-LEDs). The array is capable of producing sample excitation pulses with a width of 777 ps (FWHM), enabling short lifetime fluorophores to be investigated. The fluorescence emission is detected by a second, vertically-opposed 16 × 4 array of SPADs fabricated in 0.35 μm high-voltage CMOS technology with in-pixel time-gated photon counting circuitry. Captured chip data are transferred to a personal computer for further processing, including histogram generation, lifetime extraction, calibration and background/noise compensation. The system was demonstrated with measurements of fluorescent colloidal quantum dot and Rhodamine samples.

A SPAD–based pixel array for the analysis of fluorescence phenomena is presented in [101]. Each 150 × 150 μm^2^ pixel integrates a single photon detector combined with an active quenching circuit and a 17-bit digital events counter. On-chip master logic provides the digital control phases required by the pixel array with a full programmability of the main timing synchronisms. The pixel exhibits an average dark count rate of 3 kcps and a dynamic range of over 120 dB in time uncorrelated operation. Time-resolved fluorescence measurements have been demonstrated by detecting the fluorescence decay of quantum-dot samples without the aid of any optical filters for excitation laser light cutoff.

An integrated CMOS sensor array for fluorescence applications which enables time-gated, time-resolved fluorescence spectroscopy is presented in [102]. The 64 × 64 array is sensitive to photon densities as low as 8.8 × 10^6^ photons/cm^2^ with 64-point averaging. Through a differential pixel design, the prototype has a measured impulse response of 800 ps.

The design and characterization of a low-noise time-resolved image sensor fabricated in a 130 nm CMOS process is reported in [103]. Each pixel within the 32 × 32 pixel array contains a low-noise single-photon detector and a high-precision TDC. The 10-bit TDC exhibits a timing resolution of 119 ps with a timing uniformity across the entire array of less than two least significant bits (LSBs). The differential non-linearity (DNL) and integral non-linearity (INL) are at ±0.4 and ±1.2 LSBs, respectively. The pixel array was fabricated with a pitch of 50 μm in both directions and with a total TDC area of less than 2000 μm^2^. The target application for this sensor is time-resolved imaging, in particular fluorescence lifetime imaging microscopy.

A 32 × 32 pixel image sensor with analog counting pixels for time-gated fluorescence lifetime detection based on SPADs is reported in [104]. The sensor, fabricated in a high-voltage 0.35 μm CMOS technology, uses an analog counting approach to minimize the area occupation of pixel electronics while maintaining a nanosecond timing resolution and shotnoise-limited operation. The all n-type field-effect transistor (nFET) pixel is formed by 12 transistors and features 25-μm pitch, achieving a 20.8% fill factor. The chip includes a phase-locked loop circuit for gating window generation, working at a maximum repetition frequency of 40 MHz. The sensor can be gated at frequency up to 80 MHz using an external delay generator. Optical characterization with a picosecond-pulsed laser showed a minimum gating window width of 1.1 ns.

## 4. Conclusions

Two characteristics are noteworthy when combining the use of UCNP with CMOS technology. First, the excitation for UCNPs is in the near-infrared band. Due to the bandgap of silicon, typical CMOS detectors have a quantum efficient of below 10% for wavelengths longer than 900 nm. Therefore, since silicon is a poor absorber of the NIR excitation, using CMOS with UCNP reduces the excitation-rejection optical filter attenuation requirement.

Second, for time-resolved measurements, the UCNPs can operate in luminescence at the micro-second time frame, which is much longer than that of fluorescence with a lifetime typically in the nano-second time frame. While modern CMOS technology is capable of operating on the nano-second time scale, operating CMOS circuits at high speeds leads to high power consumption. High-speed operation can be achieved with acceptable power consumption with the use of modern, highly scaled CMOS processes. However, this leads to high fabrication cost and is especially not suitable for the low-volume analytical instrumentation market (as opposed to high-volume consumer electronics). Therefore, in this respect, employing UCNPs with CMOS offer substantial power advantage, which translates to reduced system complexity and cost.

The synergy between UCNPs and CMOS is currently a new concept. As a result, experimental prototypes are not widely demonstrated. By highlighting key characteristics and advantages, this paper is intended to stimulate further research in this emerging area.

## Figures and Tables

**Table 1.** UC emissions wavelengths from lanthanide-doped UCNPs with varying host overall compositions and structures. The variability in emissions profiles exhibited by similar systems exemplifies their optical tunability.

**Dopant Ions**				**Major Emissions (nm)**			

**Host Lattice**	**Sensitizer**	**Activator**	**Shell**	**Blue**	**Green**	**Red**	**Ref.**

***Composition Tuning***							

α-NaYF_4_	20% Yb	2% Er		411	540	660	[4]
	20% Yb	0.2% Tm		450, 475		647	[6]
	20% Yb	0.2% Er, 0.2% Tm		499, 474	525	644, 693	[5]

β-NaYF	20% Yb	2% Er			520, 540		[7]
	20% Yb	0.2% Tm		450, 476		654	
	20% Yb	2% Ho			541		

β-NaYF_4_	18.6%Yb	2.2% Er	SiO_2_	407	521, 539	651	[8]
	25% Yb	0.3% Tm	SiO_2_	450, 479			

β-NaYF_4_	18% Yb	2% Er		410		650	[5]
	60% Yb	2% Er			520, 545	650	
	20% Yb	2% Tm		450, 476	545	800 (NIR)	
	20% Yb	0.2% Tm, 1.5% Er		410,		650	
	20% Yb	0.2% Tm, 0.2%		450, 476	520, 545	650	

β-NaYF_4_	20% Yb	0.2% Tm		452, 476			[9]
	20% Yb	10% Tm		452			

NaGdF_4_	20% Yb	0.2% Er			520, 540	664	[10]
	20% Yb	0.2% Ho			538	644	
	20% Yb	0.2% Tm		450, 477		649	
	20% Yb	0.2% Tm, 0.2% Ho		450, 477	538	644	
	20% Yb	0.2% Tm, 1% Ho			538	644	

YF_3_	10% Yb	0.5% Er			521, 543	660	[11]
	10% Yb	0.2% Tm		360, 450, 476		650, 700	
	10% Yb	0.5% Er, 0.2%		380, 410, 450, 476	521, 543	650	

LiYF_4_	18% Yb	2% Er		410	525, 540	660	[12]
	20% Yb	0.2% Tm		475		650	
	18% Yb	0.2% Ho			525	650	
	20% Yb	0.2% Tm, 1% Er		475	525, 540	650, 660	
	20% Yb	0.2% Tm, 0.5% Ho		475	540	660	

Y_2_O_3_	10% Yb	1% Er			535, 555, 565	660	[13]
	1% Yb	1% Er				660	

***Cross-Relaxation Tuning***							

α-NaYF	20% Yb	2% Ho			541	647, 750	[14]
α-NaYF_4_	20% Yb	2% Ho, 15% Ce				647, 750	

β-NaYF_4_	20% Yb	5% Er			544	657	[15]
β-NaYF_4_	20% Yb	5% Er, 25% Mn				657	

β-NaYF_4_	20% Yb	0.5% Tm		410	545	650	[10]
β-NaYF	20% Yb	0.5% Tm, 80% Li			545		

***Core-Shell Tuning***							

β-NaYF	20% Yb	0.3% Tm	β-NaYF : 20% Yb, 2%Er	410, 450, 475	525, 540	660	[16]

β-NaGdF_4_	49% Yb	1% Tm	β-NaGdF_4_: 15% Tb	452, 476, 490	545, 560		[17]
	49% Yb	1% Tm	β-NaGdF_4_: 15% Eu	452, 476	595	625	
	49% Yb	1% Tm	β-NaGdF_4_: 15% Dy	452, 476	575	615, 690	
	49% Yb	1% Tm	β-NaGdF_4_: 15% Sm	452, 476	555, 595		

β-NaYF	18.6% Yb	2.2% Er	SiO_2_ -TRITC	407		651	[8]
	25% Yb	0.3% Tm	SiO_2_ -FITC	450, 479	521, 539, 580		
	25% Yb	0.3% Tm	SiO_2_-QD_605_	450, 479	540	605	

β-NaYF_4_	20% Yb	2% Tm	Rhodamine B			660	[18]
β-NaYF_4_	20% Yb	2% Tm	Dye S-0378				
β-NaYF_4_	20% Yb	3.5% Er	Fluorescein		525, 540	802 (NIR)	
β-NaYF_4_	20%Yb	3.5% Er	Dye NIR-797	475			

β-NaYF_4_	20% Yb	2% Er	QD_555_		580	660	[19]

**Table 2.** Lifetimes of different lanthanide-doped host lattices. The reader is referred to references presented for dopant concentrations and synthetic methods. Dopants on same line indicate co-doping.

**Host Lattice**	**Dopants**	**Lifetime (ms)**	**Reference**
LaPO_4_	Ce^3+^, Tb^3+^	3.08Tb^3+^	[49]

NaYF_4_	Ce^3+^, Tb^3+^	2.21Tb^3+^	[57]

KGdF_4_	Eu^3+^	10.34	[58]
	Tb^3+^	9.48	
	Dy^3+^	1.58	

CaF_2_	Ce^3+^, Tb^3+^	12.5Tb^3+^	[59]

ZrO_2_	Tb^3+^	1.82	[60]
